# Impact of the SARS-CoV-2 pandemic on the survival of patients with high-grade glioma and best practice recommendations

**DOI:** 10.1038/s41598-023-29790-8

**Published:** 2023-02-16

**Authors:** Marco M. E. Vogel, Arthur Wagner, Jens Gempt, Harald Krenzlin, Thomas Zeyen, Richard Drexler, Martin Voss, Charlotte Nettekoven, Tammam Abboud, Dorothee Mielke, Veit Rohde, Marco Timmer, Roland Goldbrunner, Joachim P. Steinbach, Lasse Dührsen, Manfred Westphal, Ulrich Herrlinger, Florian Ringel, Bernhard Meyer, Stephanie E. Combs

**Affiliations:** 1 Department of Radiation Oncology, Klinikum rechts der Isar, School of Medicine, Technical University of Munich (TUM), Ismaninger Strasse 22, 81675 Munich, Germany; 2 Institute for Radiation Medicine (IRM), Department of Radiation Sciences (DRS), Helmholtz Zentrum München, Ingolstädter Landstrasse 1, 85764 Neuherberg, Germany; 3 Department of Neurosurgery, Klinikum rechts der Isar, School of Medicine, Technical University of Munich (TUM), Ismaninger Strasse 22, 81675 Munich, Germany; 4 Department of Neurosurgery, University Medical Center Mainz, Langenbeckstrasse 1, 55131 Mainz, Germany; 5 Division of Clinical Neurooncology, Department of Neurology and Center of Integrated Oncology, University Hospital Bonn, Rheinische Friedrich-Wilhelms-University of Bonn, Venusberg-Campus 1, 53105 Bonn, Germany; 6 Department of Neurosurgery, University Medical Center Hamburg-Eppendorf, Universität Hamburg, Martinistrasse 52, 20246 Hamburg, Germany; 7 Dr. Senckenberg Institute of Neurooncology, University Hospital Frankfurt, Goethe University, Schleusenweg 2-16, 60528 Frankfurt/Main, Germany; 8 Center for Neurosurgery, Faculty of Medicine, University Hospital Cologne, University of Cologne, Kerpener Strasse 62, 50937 Cologne, Germany; 9 Department of Neurosurgery, University Medical Center Göttingen, University of Göttingen, Robert-Koch-Strasse 40, 37075 Göttingen, Germany; 10 Deutsches Konsortium Für Translationale Krebsforschung (DKTK), Partner Site Munich, Munich, Germany

**Keywords:** Outcomes research, CNS cancer

## Abstract

The severe acute respiratory syndrome coronavirus type 2 (SARS-CoV-2) has changed the clinical day-to-day practice. The aim of this study was to evaluate the impact of the pandemic on patients with high-grade glioma (HGG) as well as to derive best practice recommendations. We compared a multi-institutional cohort with HGG (n = 251) from 03/2020 to 05/2020 (n = 119) to a historical cohort from 03/2019 to 05/2019 (n = 132). The endpoints were outcome (progression-free survival (PFS) and overall survival (OS)) as well as patterns of care and time intervals between treatment steps. The median OS for WHO grade 4 gliomas was 12 months in 2019 (95% Confidence Interval 9.7–14.3 months), and not reached in 2020 (*p* = .026). There were no other significant differences in the Kaplan–Meier estimates for OS and PFS between cohorts of 2019 and 2020, neither did stratification by WHO grade reveal any significant differences for OS, PFS or for patterns of care. The time interval between cranial magnetic resonance imaging (cMRI) and biopsy was significantly longer in 2020 cohort (11 versus 21 days, *p* = .031). Median follow-up was 10 months (range 0–30 months). Despite necessary disease containment policies, it is crucial to ensure that patients with HGG are treated in line with the recent guidelines and standard of care (SOC) algorithms. Therefore, we strongly suggest pursuing no changes to SOC treatment, a timely diagnosis and treatment with short time intervals between first symptoms, initial diagnosis, and treatment, as well as a guideline-based cMRI follow-up.

## Introduction

After 2020, when the world first faced the new severe acute respiratory syndrome coronavirus type 2 (SARS-CoV-2)^1^, the clinical day-to-day practice for virtually any health care provider changed severely. The rapidly rising numbers of cases with increasing admissions to the intensive care units held the health care system hostage and led to changes in patterns of care for cancer patients^2^. This might have an impact on the oncologic outcome of cancer patients. Patients with high-grade glioma (HGG), whose survival may be drastically reduced, are particularly vulnerable during pandemic times, when the focus of health care providers shifts to containing and treating the contagion.

Previously, Azab et al. published a first evidence-based review on the impact of SARS-CoV-2 on the management of glioma patients^3^. The authors found no significant difference between SARS-CoV-2 negative and positive patients concerning surgical admissions. Complications and mortality outcomes were more significant in negative than positive patients. However, the authors reported the data to be heterogeneous.

Back in May 2020, we reported first practice recommendations on the neuro-oncology management for patients with HGG during the SARS-CoV-2 pandemic^4^. With the knowledge at that time, we proposed scenarios in cases of a possible pandemic crisis including hypofractionated radiotherapy (RT), modified chemotherapy to minimize immunosuppression, and in some cases omission of treatment if patients tested positive for SARS-CoV-2^4^. However, with the growing evidence best practice recommendations might look different nowadays. Therefore, the aim of this article is to evaluate the actual impact of the SARS-CoV-2 pandemic on the outcome and patterns of care for patients with HGG, as well as to derive best practice recommendations for similar future scenarios.

## Material and methods

### Patients

We established a multi-institutional, retrospective and anonymized database, and collected data from seven German tertiary care centers specialized in neuro-oncology (*Klinikum rechts der Isar of the Technical University of Munich (TUM), University Hospital Frankfurt am Main, University Medical Center Hamburg-Eppendorf, University Hospital Bonn, University Medical Center Göttingen, University Hospital Cologne, University Medical Center Mainz*). We included patients with HGG (WHO grades 3 and 4) treated or diagnosed between March 2020 and May 2020, which comprised the primary study group. A historical control group was accrued from the corresponding period in 2019. The institutional review board of the Technical University of Munich (TUM) approved the study (No. 676/20 S). We performed this analysis in compliance with the principles of the Declaration of Helsinki and its latter amendments^5^. The need for informed consent was waived under the Bavarian Hospital Law (Bayerisches Krankhausgesetz Art. 27 Abs. 4) due to the study’s retrospective and anonymous design. The study is part of the egePan Unimed consortium which is funded by the Bundesministerium für Bildung und Forschung (BMBF) with the identifier 01KX2021. All data were anonymized before transferal between centers and storage. Only study personnel received access to data.

### Endpoints

The primary endpoints were the estimations of overall survival (OS) and progression-free survival (PFS). Secondary endpoints were OS and PFS at 6 and 12 months. Further, we exploratively evaluated the impact of the SARS-CoV-2 pandemic on the patterns of care (e.g. time intervals between treatment steps).

### Statistical analysis

The patient cohorts were compared by Kaplan–Meier estimators of OS and PFS with log rank testing for statistically significant differences. The predetermined times of 6 and 12 months for both OS and PFS were analyzed by z-tests. Student’s t-tests were used to compare metric baseline parameters.

All other statistical analyses were performed descriptively with exploratory intention using proportions, means, medians, and 95%-confidence intervals (95%CI). All statistical analyses were performed with SPSS version 28 (IBM, Armonk, USA). A p value of less than 0.05 was defined as statistically significant.

## Results

Over 7 German university hospitals, 251 patients with a median age of 61 years (range 18–88 years) were included. 119 patients were treated in 2020, while 132 patients were treated in 2019. Patient characteristics are depicted in Table 1. In 2019, data on follow-up was available for 80% of grade 3 gliomas after 12 months, and in 2020 for 87.5% after 12 months. For grade 4 gliomas, 86.9% of the follow-up data were available after 12 months in 2019, and 78.6% in 2020. Two patients (1.7%) with grade 4 gliomas in the 2020 cohort tested positive for SARS-CoV-2 during the follow-up regimen, although without any impact on scheduling.

**Table 1:** Patient Characteristics of the cohorts from 2019 and 2020.

		Treatment year			P
		Total (n = 251)	2019 (n = 132)	2020 (n = 119)	
Age in years, mean ± SD		61.3 ± 12.8	61.0 ± 11.8	61.6 ± 13.3	.732
Median (range)		61 (18–88)	61 (18–88)	63 (21–87)	
Gender	Female	105	52	53	.409
		41.8%	39.4%	44.5%	
	Male	146	80	66	
		58.2%	60.6%	55.5%	
Entity	Anaplastic astrocytoma	9	1	8	.260
		3.6%	0.8%	6.7%	
	Anaplastic oligodendroglioma	17	9	8	
		6.8%	6.8%	6.7%	
	Glioblastoma	217	118	99	
		86.4%	89.4%	83.2%	
	Gliosarcoma	2	1	1	
		0.8%	0.8%	0.8%	
	Medulloblastoma	2	1	1	
		0.8%	0.8%	0.8%	
	Other	4	2	2	
		1.6%	1.5%	1.6%	
WHO	3	26	10	16	.128
		10.4%	7.6%	13.4%	
	4	225	122	103	
		89.6%	92.4%	86.6%	
IDH mutation	Mutated	28	13	15	.489
		11.2%	9.8%	12.6%	
	Wildtype	221	118	103	
		88.0%	89.4%	86.6%	
	Unknown	2	1	1	
		0.8%	0.8%	0.8%	
MGMT promotor	Negative	123	65	58	.537
		49.0%	49.2%	48.7%	
	Positive	124	66	58	
		49.4%	50.0%	48.7%	
	Unknown	4	1	3	
		1.6%	0.8%	2.6%	
1p19q codeletion	Co-deleted	17	8	9	.455
		6.8%	6.1%	7.6%	
	Non-co-deleted	61	28	33	
		24.3%	21.2%	27.7%	
	Unknown	173	96	77	
		68.9%	72.7%	64.7%	

WHO—World Health Organization; IDH—isocitrate dehydrogenase; MGMT—O6-Methylguanine-DNA Methyltransferase; KPS—Karnofsky Performance Status Scale. n = number; SD—standard deviation.

### Oncological outcome

We evaluated the OS and PFS at 6 and 12 months for patients with WHO grade 3 (see Table 2), WHO grade 4 (see Table 3), and the entire study population (see Table 4). There were no statistically significant differences in PFS at 6 or 12 months between treatment years for the entire study population with WHO grade 3 and 4 tumors. Likewise, neither did stratification by WHO grade 3 or 4 result in significant differences for any of the parameters (Tables 2 and 3).

**Table 2:** Overall survival and progression-free survival at 6 and 12 months compared between the 2019 and 2020 cohorts of Patients with WHO Grade 3 gliomas according to Kaplan–Meier estimation.

WHO 3	Treatment year			P
	Total	2019	2020	
OS-6 (n; %)	21 (95.7)	8 (100.0)	13 (93.3)	.502
OS-12 (n; %)	16 (90.6)	7 (100.0)	9 (85.6)	.329
PFS-6 (n; %)	18 (95.0)	6 (100.0)	12 (92.3)	.452
PFS-12 (n; %)	15 (87.7)	6 (100.0)	9 (79.1)	.142

OS—overall survival, PFS—progression-free survival, n—number.

**Table 3:** Overall survival and progression-free survival at 6 and 12 months compared between the 2019 and 2020 cohorts of Patients with WHO Grade 4 gliomas according to Kaplan–Meier estimation.

WHO 4	Treatment year			P
	Total	2019	2020	
OS-6 (n; %)	140 (75.2)	78 (74.0)	62 (76.6)	.219
OS-12 (n; %)	97 (54.9)	55 (48.0)	42 (66.3)	.058
PFS-6 (n; %)	140 (88.9)	78 (90.5)	62 (86.9)	.426
PFS-12 (n; %)	97 (51.7)	54 (56.6)	43 (43.9)	.095

OS—overall survival, PFS—progression-free survival, n—number.

**Table 4:** Overall survival and progression-free survival at 6 and 12 months compared between the 2019 and 2020 cohorts of all patients (WHO grade 3 and 4) according to Kaplan–Meier estimation.

WHO 3 & 4	Treatment year			P
	Total	2019	2020	
OS-6 (n; %)	141 (77.4)	77 (75.8)	64 (79.3)	.489
OS-12 (n; %)	109 (58.8)	53 (51.6)	56 (69.4)	.071
PFS-6 (n; %)	155 (89.6)	87 (91.2)	68 (87.8)	.412
PFS-12 (n; %)	95 (55.5)	57 (59.7)	38 (49.1)	.113

OS—overall survival, PFS—progression-free survival, n—number.

**Figure 1:**
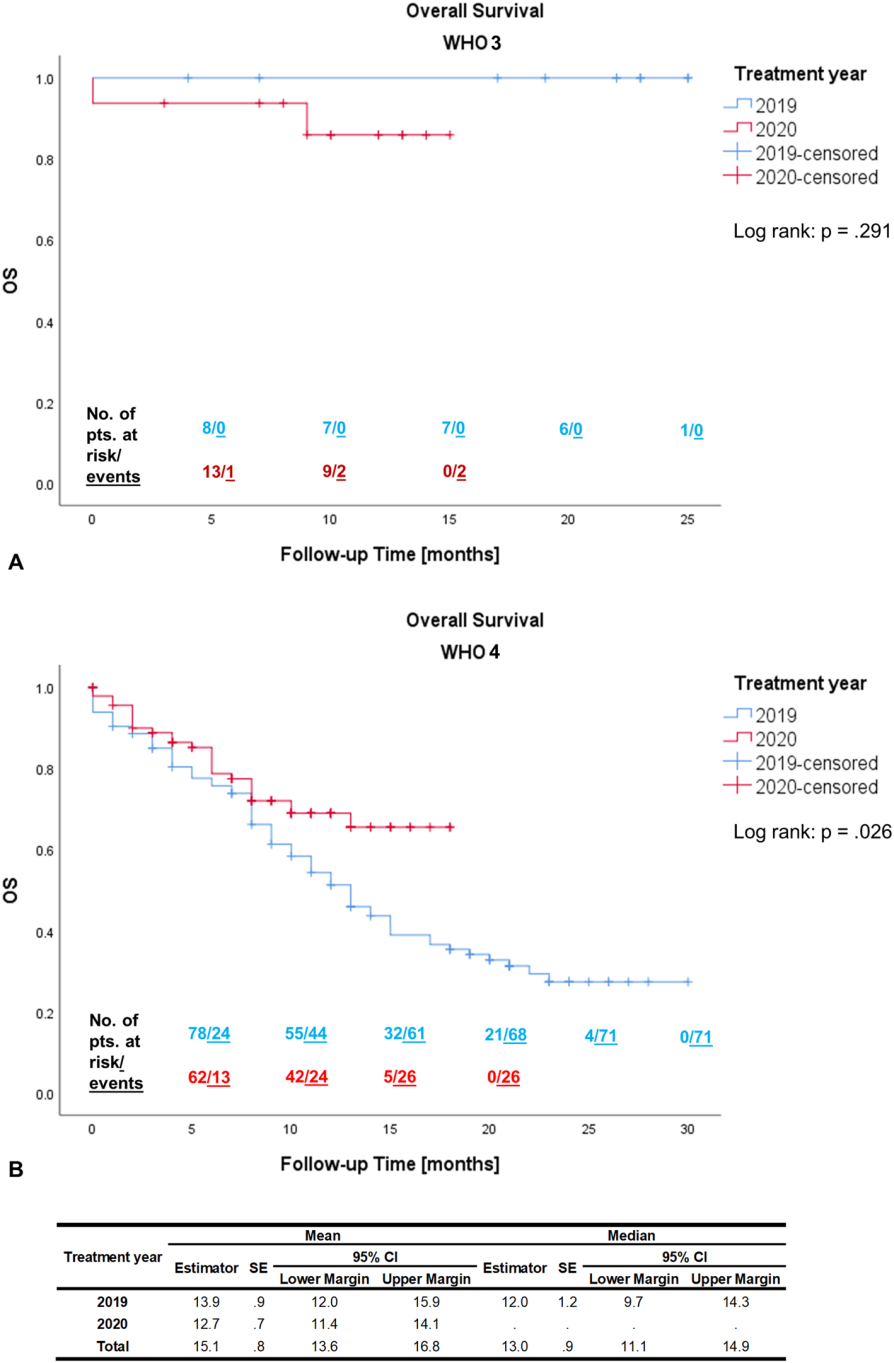
Kaplan–Meier estimator for Overall Survival (OS) compared between the 2019 and 2020 cohorts with WHO Grade 3 (**A**) and WHO Grade 4 (**B**) gliomas. Pts.—patients; SE—standard error; 95%CI—95% confidence interval. Kaplan–Meier statistics not conducted for WHO 3 due to censoring of all 2019 cases.

**Figure 2:**
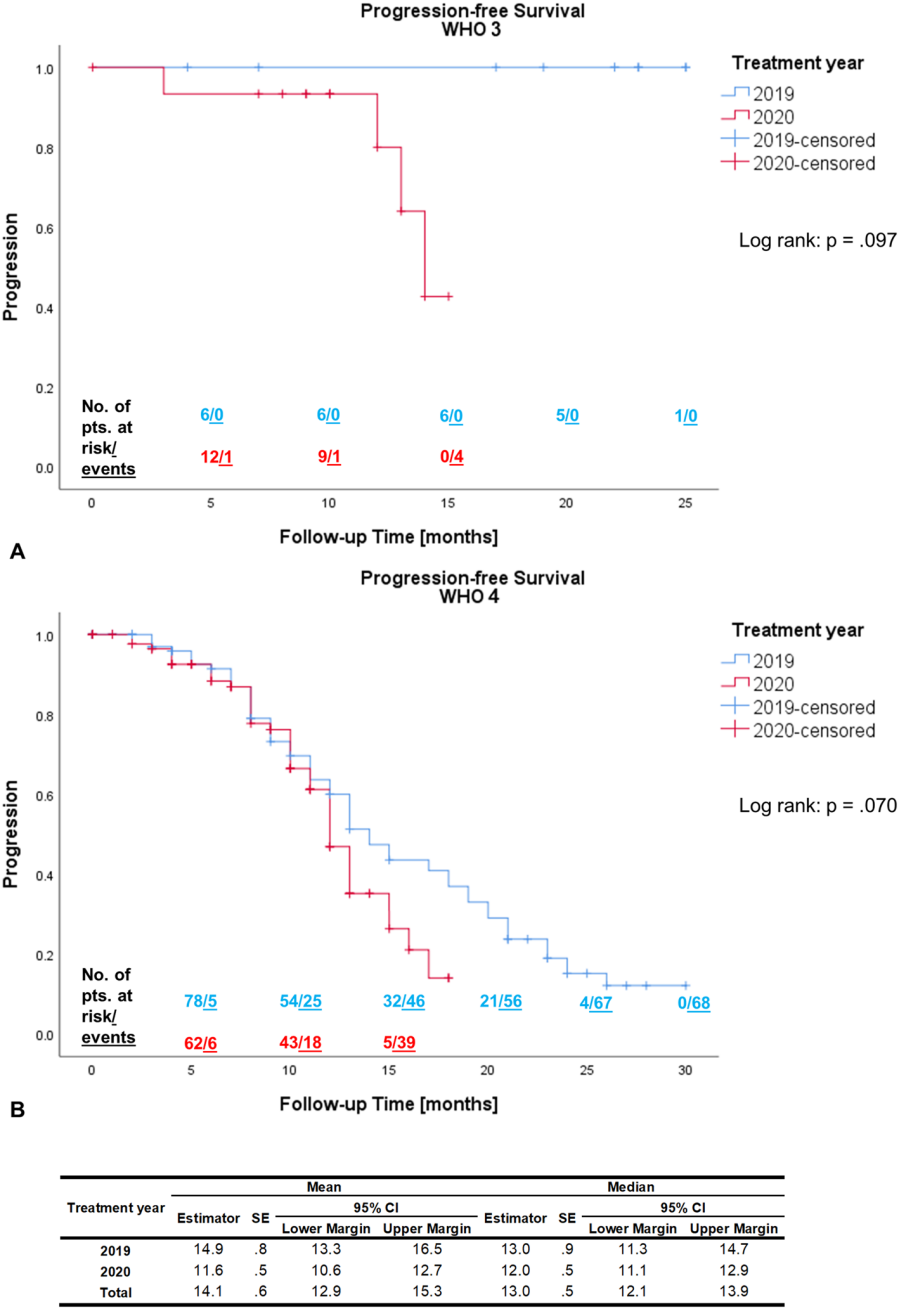
Kaplan–Meier estimator for progression-free survival (PFS) compared between the 2019 and 2020 cohorts with WHO Grade 3 (**A**) and WHO Grade 4 (**B**) gliomas. Pts.—patients; SE—standard error; 95%CI—95% confidence interval. Kaplan–Meier statistics not conducted for WHO 3 due to censoring of all 2019 cases.

The Kaplan–Meier survival analyses did not exhibit any statistically significant difference in OS for WHO 3 gliomas between 2019 and 2020 (log rank; *p* = 0.291; see Fig. 1A). The median OS for WHO grade 4 gliomas was 12 months (95%CI 9.7–14.3 months) in 2019, and not reached in 2020 (log rank; *p* = 0.026; see Fig. 1B). The estimated PFS did not differ for either WHO grade 3 (log rank; *p* = 0.097; see Fig. 2A) or 4 gliomas (log rank; *p* = 0.070; see Fig. 2B).

### Patterns of care

We further evaluated the patterns of care for patients in 2020 versus 2019. Table 5 shows a comparison of the procedures performed. There was no significant difference in the distribution between years for any of the treatment modalities.

**Table 5:** Procedures performed in comparison between the 2019 and 2020 cohorts.

		Treatment Year			P
		Total (n = 251)	2019 (n = 132)	2020 (n = 119)	
Initial resection performed (n; %)		182 (72.5)	91 (68.9)	91 (76.5)	0.176
GTR (n; %)		173 (68.9)	93 (70.5)	80 (67.2)	0.126
Adjuvant radiochemotherapy	Concomitant radiochemotherapy (n; %)	209 (83.2)	109 (82.6)	100 (84.1)	0.728
	No radiotherapy (n; %)	31 (12.4)	18 (13.6)	13 (10.9)	
	Unknown (n; %)	11 (4.4)	5 (3.8)	6 (5.0)	
Adjuvant systemic therapy	Adjuvant systemic therapy (n; %)	165 (65.7)	92 (69.7)	73 (61.4)	0.154
	No systemic therapy (n; %)	48 (19.1)	25 (18.9)	23 (19.3)	
	Unknown (n; %)	38 (15.2)	15 (11.4)	23 (19.3)	
Tumor treating fields	Received (n; %)	12 (4.7)	7 (5.3)	5 (4.2)	0.864
Available follow-up with cMRI (n; %)		142 (56.5)	79 (59.8)	63 (52.9)	0.431
KPS preoperative, mean ± SD		85 ± 13.5	85 ± 13.7	84 ± 13.3	0.698
Median (95%CI)		90 (60–100)	90 (60–100)	90 (60–100)	
KPS postoperative, mean ± SD		81 ± 16.1	84 ± 14.5	78 ± 17.0	**0.039**
Median (95%CI)		80 (50–100)	90 (50–100)	80 (50–100)	
KPS at last follow-up, mean ± SD		58 ± 30.4	53 ± 30.9	64 ± 28.7	**0.004**
Median (95%CI)		60 (30–100)	60 (30–100)	70 (40–100)	
Follow-up time in months, mean ± SD		10.4 ± 6.9	11.9 ± 8.1	8.8 ± 4.7	** < .001**
Median (95%CI)		10 (1–25)	11 (1–25)	10 (1–24)	

GTR—gross total resection; cMRI—cranial magnetic resonance imaging. SD—standard deviation; 95%CI—95% confidence interval.

Significant values are in [bold].

Table 6 presents the time intervals between the treatment steps. The time interval between initial cMRI and biopsy was significantly longer for patients in the 2020 cohort (Student’s t-test; *p* = 0.031). All other time intervals showed no significant difference.

**Table 6:** Time expedited in days between treatment steps in comparison between the 2019 and 2020 cohorts.

	Treatment year			P
	Total	2019	2020	
Days between initial cMRI and resection, mean ± SD	16 ± 24	15 ± 24	18 ± 25	.441
Days between biopsy and resection, mean ± SD	28 ± 30	27 ± 16	29 ± 40	.866
Days between initial cMRI and biopsy*, mean ± SD	15 ± 22	11 ± 10	21 ± 32	**.031**
Days between resection and first RT, mean ± SD	34 ± 12	33 ± 11	35 ± 12	.426
Days between biopsy and first RT, mean ± SD	36 ± 22	37 ± 25	34 ± 19	.621
Days between last RT and first ST, mean ± SD	43 ± 93	35 ± 25	53 ± 134	.299

cMRI—cranial magnetic resonance imaging; RT—radiotherapy; ST—systemic treatment; SD—standard deviation;

*initial biopsy rate 2019: 31.3%; 2020: 23.8%.

Significant values are in [bold].

## Discussion

The diagnostics and patterns of care for HGGs rely not only on the intricate interdisciplinary cooperation within the infrastructure of a tertiary care center^6–8^. Patients are also dependent on timely referrals by the out-patient sectors of health care systems, necessitating well-organized communication and scheduling structures to ensure early histological diagnosis and rapid initiation of adjunct treatment regimens^9^. Our data demonstrated that when patients entered care at specialized centers, timely surgery and radiation were offered and no delay in treatment was observed.

Delays within these tried and tested frameworks may result in a subpar oncological outcome, although there are conflicting verdicts on this in the few studies published^9–13^. In our study, it was of prime interest to assess this possible *collateral damage* of the SARS-CoV-2 pandemic by comparing a cohort within the supposedly most critical period of the pandemic during early 2020 with a historical control. Within the confines of this study, we are able to report that with the exception of the OS for grade 4 tumors, no significant decline in oncological outcome was observed. Seeing as all tumor characteristics, GTR rates and treatment strategies did not differ between these cohorts, this difference in OS may be accounted for by the fact that the historical group had significantly longer and more consistent follow-up available, which is mirrored in the Kaplan–Meier estimation. There were no other statistical differences in oncological outcome. However, the time between cMRI and biopsy was significantly longer in patients in 2020, which might indicate a delayed patient referral to the tertiary care centers.

Our gross tumor resection (GTR) rates, OS and PFS estimates are congruent with contemporary pre-pandemic data^14^. In a recent pooled meta-analysis of 19 studies, Gandhi et al. found the OS ranging between 10 to 22 months and the PFS between 5 to 11 months^15^.

Patient management and referral processes are inevitably strained by the disease-containment measures, which in turn require a different approach to triage for the handling of routine and emergency health care services. While several scientific efforts have been made to define routines that accommodate disease-containment measures with entity-specific guidelines, it is imperative for any tertiary care provider to establish a patient management framework tailored to regional public health policies as well as the current SARS-CoV-2 load^16–20^.

Our assumption holds true that integrating disease containment policies without delaying neurooncological schedules is feasible and mainly a product of organizational processes. However, some degree of *collateral damage* may be inevitable. One must note that an essential component of guideline-conform diagnostic and treatment regimens strongly depend on patient compliance, which is substantially influenced by the perception of the perceived threat of an infection with the SARS-CoV-2 pathogen. This remains a largely unstudied element, and it is difficult to gauge the extent of a possible deficit in oncological outcome for lack of quality data.

What can be gleaned from this pandemic for future similar scenarios? We strongly advocate to not altering the standard of care (SOC) paradigms of both primary and tertiary health care providers. Various articles report on a reduced outcome when patients were treated without maximal resection^21^, without radio(chemo)therapy^22,23^, without adjuvant chemotherapy^22^, or without maintenance therapy with tumor treating fields^24^. From the start, patients with HGG are vulnerable patients with reduced oncological outcome. Therefore, one should not deprive those patients of their right for treatment and the best possible outcome.

Further, a timely diagnosis and short time intervals between treatment steps are of essence. We found a significantly longer time interval between initial cMRI and biopsy during 2020. It is possible that due to the low number of cases with biopsy, the effect on the outcome might not be significant. However, it is clear that such delays during all treatment steps should be strictly avoided. Previously, Aggarwal et al. showed that patients with HGG paradoxically had a significantly worse OS when they were diagnosed earlier^12^. However, the authors compared patients with earlier diagnosis who were admitted to the hospital presenting as emergencies compared to patients with delayed diagnosis who were referred as outpatients. For delayed surgery, Flanigan et al. showed that a longer wait time to surgery was associated with a worse OS in glioblastoma patients presenting with seizures only^25^. Although there are conflicting data concerning delays between surgery and radiochemotherapy, Buszek et al. recently showed that glioblastoma patients with GTR and a postponement of > 8 weeks exhibit a worse OS^26^.

Therefore, we recommend a timely diagnosis and treatment with short time intervals between first symptoms, initial diagnosis, and treatment. To accomplish that and not jeopardize patient care, established hygiene standards and prevention interventions are extremely important. Ngandu et al. showed in their scoping review that with non-pharmaceutical prevention interventions it was possible to avoid hospital-acquired SARS-CoV-2 infections completely or in large part^27^.

Therefore, the actions taken against the pandemic remain a key factor for the fight against SARS-CoV-2, however, the measures should not lead to a delayed treatment of cancer patients, specifically patients with HGG.

The available cMRI follow-up was not significantly different in both groups, with 59.8% in 2019 versus only 52.9% in 2020. There is no existing evaluation of the effectiveness of the clinically used imaging schedules for the follow-up of patients with HGG^28^. However, we believe that a close monitoring with cMRI as described in most clinical guidelines^29,30^ is desirable, particularly in a pandemic scenario. Therefore, we suggest to closely follow patients with cMRI according to established guidelines. Follow-up of patients with HGG should not be neglected during a pandemic and this adherence to follow-up consultations must remain at high priority without violating the disease containment measures during a pandemic.

In concordance with our recent consensus paper^4^, we may collate the following best practice recommendations for the neuro-oncological management of patients with HGG during a global pandemic scenario in Table 7.

**Table 7:** Best practice recommendations for the treatment of patients with high-grade gliomas during a pandemic.

No changes in standard of care (SOC)
Timely diagnosis and treatment upholding short time intervals between first symptoms, initial diagnosis, and therapeutic strategies
Guideline-based follow-up including cranial magnetic resonance imaging
Disease containment measures as per local regulations

As it is natural with retrospective observations, our study has limitations. The median follow-up is relatively short, and a future analysis with longer follow-up is planned. Due to the shorter follow-up in the cohort of 2020, we opted to additionally calculate binary outcome analyses (PFS and OS at 6 and 12 months). It is crucial to understand that various other confounders may factor into the outcome deficit; despite similar treatment regimens, patients may avoid consultations for fear of contracting the virus, reschedule interventions, treatment, or follow-up consultations.

## Conclusion

The current pandemic demonstrated that patients with HGG are particularly vulnerable in these times. In our retrospective analysis, the Kaplan–Meier survival analyses did not exhibit any statistically significant differences in OS for WHO 3 gliomas between 2019 and 2020 (*p* = 0.291). The median OS for WHO grade 4 gliomas was 12 months (95%CI 9.7–1.43 months) in 2019, and not reached in 2020 (*p* = 0.026). The estimated PFS did not differ for either WHO grade 3 (*p* = 0.097) or 4 gliomas (*p* = 0.070). Therefore, it is essential to ensure that these patients’ treatment complies with the recent guidelines and SOC algorithms. Measures must be taken that even in times were resources need to be allocated such as during a pandemic, oncologic patient care is secured. It is important to note that some governmental disease containment policies may result in collateral damage to those in need of regular health care, which holds especially true for HGG patients when patient referrals and follow-up schedules are not secured. In summary, we suggest applying no changes to SOC treatment and pursuing short time intervals between first symptoms, initial diagnosis, therapeutic strategies, as well as cMRI follow-up according to established guidelines.

## Supplementary Information

Supplementary Information.

## Data Availability

The datasets generated during and analyzed during the current study are available from the corresponding author on reasonable request.
