# Supplementary material for: Impact of the SARS-CoV-2 pandemic on the survival of patients with high-grade glioma and best practice recommendations
Source: Sci Rep. 2023 Feb 16;13:2766. doi: 10.1038/s41598-023-29790-8 (PMC9933015; doi:10.1038/s41598-023-29790-8)
Supplement: Supplementary file 1 — Supplementary Information. [file 41598_2023_29790_MOESM1_ESM.docx]

**Supplementary Information**

**Supplementary Table S1** Cox regression model of the predictors age at presentation and gender for overall survival (OS).

|  | **df** | **P** | **Exp (B)** | **95% CI** |
| --- | --- | --- | --- | --- |
| **Age at presentation** | 1 | **< .001** | 1.038 | 1.021 – 1.054 |
| **Gender** | 1 | .381 | 0.834 | 0.556 – 1.252 |

df – degrees of freedom; P – level of significance; Exp (B) –
Proportional hazard coefficient; 95% CI – 95% confidence
interval.

**Supplementary Table S2** Descriptive statistics of metric parameters in dataset.

|  | **Minimum** | **Maximum** | **Mean** | **SE** | **SD** | | **Variance** | **Kurtosis** | **SE** |
| --- | --- | --- | --- | --- | --- | --- | --- | --- | --- |
| **Age at presentation** | 18 | 88 | 61.28 | .918 | | 12.834 | 205.463 | .305 | .310 |
| **Follow-up [months]** | 0 | 30 | 10.44 | .455 | | 6.940 | 48.164 | -.305 | .318 |
| **OS [months]** | 0 | 90 | 17.36 | 1.352 | | 19.264 | 371.114 | 4.567 | .340 |
| **PFS [months]** | 0 | 30 | 8.46 | .429 | | 6.130 | 37.579 | 1.221 | .339 |
| **Days between inital cMRI and Resection** | 0 | 198 | 15.65 | 1.845 | | 24.461 | 598.835 | 28.190 | .364 |
| **Days between Biopsy and Resection** | 0 | 124 | 27.88 | 7.335 | | 30.243 | 914.610 | 5.975 | 1.063 |
| **Days between inital cMRI and Biopsy** | 0 | 128 | 14.84 | 2.437 | | 22.205 | 493.061 | 11.757 | .523 |
| **Days between Resection and first RT** | 10 | 78 | 34.00 | .968 | | 11.655 | 135.833 | .918 | .400 |
| **Days between Biopsy and first RT** | 7 | 131 | 35.98 | 2.900 | | 22.436 | 504.729 | 5.975 | .608 |
| **Days between last RT and first ST** | 0 | 127 | 43.41 | 8.780 | | 93.332 | 8710.851 | 100.453 | .451 |
| **Karnofsky-Index preoperative** | 20 | 100 | 85.13 | .966 | | 13.484 | 181.813 | 3.657 | .346 |
| **Karnofsky-Index postoperative** | 20 | 100 | 81.06 | 1.147 | | 16.134 | 260.291 | 1.406 | .344 |
| **Karnofsky-Index at Last Follow-up** | 0 | 100 | 57.48 | 2.079 | | 30.415 | 925.058 | -.610 | .331 |

OS – overall survival; PFS – progression-free survival; cMRI – cranial magnetic resonance imaging; RT – radiotherapy; ST – systemic treatment; SE – standard error; SD – standard deviation.
